# Assessing the effectiveness of imidacloprid and thiamethoxam via root irrigation against *Megalurothrips usitatus* (Thysanoptera: Thripidae) and its residual effects on cowpea

**DOI:** 10.1093/jee/toad166

**Published:** 2023-08-31

**Authors:** Xiao-Rui Yu, Talha Tariq, Ling-Hang Guo, Sheng-Yong Wu, Liang-De Tang, Lian-Sheng Zang

**Affiliations:** National Key Laboratory of Green Pesticide, Key Laboratory of Green Pesticide and Agricultural Bioengineering, Ministry of Education, Center for R&D of Fine Chemicals of Guizhou University, Guiyang 550025, PR China; National Key Laboratory of Green Pesticide, Key Laboratory of Green Pesticide and Agricultural Bioengineering, Ministry of Education, Center for R&D of Fine Chemicals of Guizhou University, Guiyang 550025, PR China; Key Laboratory of Integrated Pest Management on Tropical Crops, Ministry of Agriculture and Rural Affairs, Environment and Plant Protection Institute, Chinese Academy of Tropical Agricultural Sciences, Haikou 571101, PR China; State Key Laboratory for Biology of Plant Diseases and Insect Pests, Institute of Plant Protection, Chinese Academy of Agricultural Sciences, Beijing 100193, PR China; National Key Laboratory of Green Pesticide, Key Laboratory of Green Pesticide and Agricultural Bioengineering, Ministry of Education, Center for R&D of Fine Chemicals of Guizhou University, Guiyang 550025, PR China; Key Laboratory of Integrated Pest Management on Tropical Crops, Ministry of Agriculture and Rural Affairs, Environment and Plant Protection Institute, Chinese Academy of Tropical Agricultural Sciences, Haikou 571101, PR China; National Key Laboratory of Green Pesticide, Key Laboratory of Green Pesticide and Agricultural Bioengineering, Ministry of Education, Center for R&D of Fine Chemicals of Guizhou University, Guiyang 550025, PR China

**Keywords:** neonicotinoid, UPLC–MS/MS, *Megalurothrips usitatus*, *Vignia unguiculata*, root irrigation application

## Abstract

Systemic neonicotinoid insecticides (NEOs) applied by seed-treatment or root application have emerged as a prevalent strategy for early-season insect pest management. This research investigated the effectiveness of imidacloprid and thiamethoxam, administered through root irrigation, in managing thrips in cowpea [*Vigna unguiculata* (Linn.) Walp.], and the residual properties of both insecticides in cowpea and soil. The results show that thrips density depends on the application rate of insecticides. At the maximum application rate (1,500 µg/ml, active ingredient), imidacloprid and thiamethoxam controlled thrips densities below the economic injury level (EIL, the EIL of thrips on cowpea was 7/flower) for 20 days and 25 days with the density of 6.90 and 6.93/flower at the end of the periods, respectively. Imidacloprid and thiamethoxam residues decreased gradually over time and decreased sharply after 15 days after treatment (DAT), the 2 insecticides were not detected (<0.001 mg/kg) at 45 DAT. According to our findings, the application of imidacloprid and thiamethoxam via root irrigation proved residual control lasting up to 20–25 days for controlling thrips damage at experimental rates, with a strong association to their residual presence in cowpea (0.6223 < *R*^2^ < 0.9545). Considering the persistence of the imidacloprid and thiamethoxam, the maximum tested application rate (1,500 µg/ml) was recommended. As the residues of imidacloprid and thiamethoxam were undetectable in cowpea pods at all tested rates, it may be suggested that the use of each insecticide is safe for consumers and effective against thrips, and could be considered for integrated thrips management in the cowpea ecosystem.

## Introduction

Cowpea [*Vigna unguiculata* (Linn.) Walp.] is an important vegetable crop rich in plant protein (Omo-Ikerodah et al. 2008), its grains and leaves for the human and livestock consumption (Agbahoungba et al. 2021), which provide a sizeable portion of human dietary protein (Jayathilake et al. 2018). It is widely grown in tropical and subtropical regions of the world (Timko et al. 2007). In Africa, cowpea is also an important major staple food crop (Agbahoungba et al. 2021, Abebe and Alemayehu 2022). Diseases and pests, particularly thrips [*Megalurothrips sjostedti* (Trybom)] (Thysanoptera: Thripidae), are the limiting factors in cowpea production, causing 20–80% losses in cowpea yield (Nabirye et al. 2003, Ngakou et al. 2008), even almost 100% yield losses were reported (Singh and Allen 1980, Agbahoungba et al. 2021). While, in other parts of the world outside Africa, the thrips species, *Megalurothrips usitatus* (Bagnall), can cause severe damage to cowpea (Tang et al. 2023) and other leguminous crops (Tang et al. 2015a), although there is currently no specific estimate of yield loss due to this species of thrips. *M. usitatus* causes damage during the entire growth period of cowpea, as it causes leaf curling at the seedling stage, growth point necrosis at the flower bud differentiation stage, flower falling at the flowering stage, and pericarp scabbing at the fruiting stage (Tang et al. 2023). Therefore, it is necessary to control this pest during the entire growth period of cowpea. In addition, *M. usitatus* can also transmit a variety of plant viruses (Prasada Rao et al. 2003, Sonali Shukla et al. 2005). Current control measures for thrips in cowpea management include cultivation of resistant varieties (Alabi et al. 2003, Abudulai et al. 2006, Omo-Ikerodah et al. 2008, Togola et al. 2019), interplanting with graminaceous crops, such as corn and sorghum (Kyamanywa and Tukahirwa 1988, Gethi and Mueke 1995, Adati et al. 2007), and physical and chemical trapping technologies (Abtew et al. 2015, Diabate et al. 2019a, 2019b, Liu et al. 2020). Biocontrol methods, such as the use of predatory insects and mites (Gitonga et al. 2002, Liu et al. 2018), pathogenic microorganisms (Wu et al. 2021), and botanical pesticides (Oparaeke et al. 2006, 2007, Oparaeke 2007), have also been implemented to control thrips during cowpea cultivation. However, chemical control is still the most efficient measure for thrips control in cowpea production due to the high activity of synthetic pesticides (Oparaeke et al. 2007).

Neonicotinoid insecticides (NEOs), with their high agonist effects on insect neuronal nicotinic acetylcholine receptors (nAChRs) (Houchat et al. 2020), are currently the most widely used insecticides in the world. In China and elsewhere, they are commonly used against piercing–sucking feeding insects, such as thrips (Meena et al. 2020). Imidacloprid is a first-generation NEO developed by Bayer AG (Germany), whereas thiamethoxam is a second-generation NEO developed by Syngenta Crop Protection (Switzerland). In China, imidacloprid and thiamethoxam have been registered as a single or combined formulation for the control of thrips in cowpea plants (China Pesticide Information Network. 2022). However, they have not met the production needs of cowpea cultivars because of their low field efficacy when applied via foliar spraying (Tang et al. 2022). Nevertheless, because these insecticides act systemically, traveling through plant tissues and protecting all parts of the crop, and then acting as neurotoxins against pests, they have been widely applied on seeds and roots of various crops for pest control (Goulson 2013). A recent study has reported the efficacy of foliar applications of thiamethoxam mixed with adjuvants on *M. usitatus* and its degradation dynamics and residues on cowpea. Adding adjuvants can significantly improve efficacy but does not affect the residual dynamics (0.121–0.223 mg/kg after 5 days treatment) (Wang et al. 2022). However, the efficacy of root irrigation application of imidacloprid and thiamethoxam on *M. usitatus* and their dissipation dynamics in the environment and plant tissue are still unknown. We hypothesized that root irrigation treatment will have a more lasting retention in cowpea plants and have a more lasting control efficacy on thrips. Thus, in this study, we investigated the uptake and translocation of imidacloprid and thiamethoxam in cowpea and their residues in the soil environment to define the relationship between detectable insecticide levels and the density of *M. usitatus* in plants. Our results broadened our understanding of the efficacy of the root irrigation application method of imidacloprid and thiamethoxam in the control of thrips in cowpea.

## Materials and Methods

### Tested Insecticides and Reagents

We used commercial formulations of imidacloprid (Admire 70% Water Dispersible Granules (WDG), Bayer Crop Science (China) Co., Ltd., Hangzhou, China) and thiamethoxam (Actara 25% WDG, Syngenta Crop Protection (Suzhou) Co., Ltd., Suzhou, China) for our experiments. The analytical standards of imidacloprid and thiamethoxam were purchased from Sigma (St Louis, Missouri, USA). Methanol and acetonitrile (MS grade) were purchased from Fisher Scientific (Pittsburgh, USA). Sodium acetate, acetic acid (MS grade), sodium acetate, and N-propyl ethyl diamine was obtained from Sigma–Aldrich (St Louis, Missouri, USA). Ultrapure water was prepared using a Milli-Q water purification system (Millipore, Bedford, MA, USA).

### Field Experiments and Sample Collection

Cowpea (variety Haoyunlai888 [Jiangxi Yuefeng Seed Industry Co., Ltd, Nanchang, China]) seeds were sown in a bed measuring approximately 600 m^2^ (30 × 20 m) at the experimental base of Hainan Academy of Agricultural Sciences, Chengmai County, Hainan Province, China, on 16 March 2020. Two lines of seeds were planted in each row. The spaces between and within the lines were 60 × 15 cm, and the distance between the adjacent rows was 50 cm. Imidacloprid and thiamethoxam were diluted to make 1500, 750, and 250 µg/ml solutions for root irrigation application. Fifty milliliters (0.075, 0.0375, and 0.0125 g a.i. for corresponding concentration, respectively) of insecticide liquid was applied per plant (hole) at the 5-leaf stage (10 days after sowing) in a randomized complete block experimental design with 3 rows (blocks) with treatments randomized within rows. All cowpea plants in a row (block) received each treatment. Each row was a treatment, and all above treatments were replicated 3 times. Cowpea plants treated with equal parts of water served as controls. The efficacy of imidacloprid and thiamethoxam, applied via root irrigation, against *M. usitatus* was evaluated at 0 (pretreatment), 5, 10, 15, 20, 25, 30, and 45 days after treatment (DAT) by sampling 10 leaves from the top, middle, and bottom of cowpea plants (a total of 30 leaves) from each experimental block. During the flowering stage, flowers (including flower buds), instead of leaves, were sampled because *M. usitatus* prefers flowers. The numbers of *M. usitatus* adults and nymphs on the leaves and flowers (buds) of randomly sampled cowpea plants in each replicate within each treatment at different growth stages were counted and statistically analyzed. In order to ensure the accuracy of counting, the leaves or flowers with thrips were removed and packed into a sealed bag and brought back to the laboratory for calculation.

Plant and soil samples were randomly collected from each of the blocks, with 3 cowpea plants collected for each treatment. Five leaves (including stems) from the top of individual cowpea plants were collected, except for the last sampling period (pods). Two hundred grams of soil were sampled from a radius of 15 cm around the root and to a depth of 15 cm (Wu et al. 2019). Plant and soil samples were collected at 5, 10, 15, 20, 25, 30, and 45 DAT and then analyzed by ultra-performance liquid chromatography–tandem mass spectrometry (UPLC–MS/MS). The collected samples were stored at −20 °C prior to analysis.

### Analytical Procedures

#### Instrumentation.

An AcQuity UPLC system (WATERS, Massachusetts, USA) coupled with a Q Exactive and an Xcalibur workstation from Thermo (USA) was used for pesticide analysis. Target compounds were separated chromatographically at 40 °C on an AcQuity Waters HSS T3 column (2.1 mm ID × 50 mm, 1.8 μm particle size, Waters, Wexford, Ireland). Gradient elution was performed with 0.1% (v/v) acetic acid-water water (A) and acetic acid-acetonitrile (B) at a flow rate of 0.3 ml/min. The injection volume for all the experiments was 2 μl. The gradient program was as follows: 0 min, 90% solvent B; 2 min, 90% solvent B; 6 min, 40% solvent B; 9 min, 40% solvent B; 9.1 min, 90% solvent B; 12 min, 90% solvent B. The total run time for each injection was 12 min. The samples were separated using a gradient that started at 10% eluent A for 2 min, increasing to 60% eluent A for 4 min, holding for 3 min, decreasing eluent A to 10% at 9.1 min, and then equilibrating for 3 min. Data were recorded on a Q Exactive hybrid Q–Orbitrap mass spectrometer equipped with a heated ESI (Electron Spray Ionization) source (Thermo Fisher Scientific, Waltham, MA, USA) utilizing the single ion monitoring mode on positive ion mode of MS. The ESI source parameters were optimized and performed as follows: the capillary voltage was 3.0 kV, sheath gas pressure was 40 arb, aux gas pressure was 10 arb, sweep gas pressure was 0 arb, the capillary temperature was 320 °C, and aux gas heater temperature was 350 °C.

#### Sample preparation.

About 250 g of soil sample and 100 g of cowpea plant (leaves and stems) sample were chopped and homogenized by high throughput tissue crusher (Tissuelyser-24L, Shanghai, China) under − 20 °C. Then, a 10 g soil and 1.50 g plant sample were mixed with 15 ml acetonitrile and vortexed for 3 min. Thereafter, 5 g magnesium sulfate was added to the solution and vortexed for 10 min. The mixture was centrifuged at 10,000×g for 10 min, and 2 ml of supernatant and 50 mg of primary−secondary amine (PSA, final concentration; 25 mg/ml) was mixed together using a vortex mixer for 3 min. After centrifugation at 10,000×g for 10 min, 1.5 ml of the supernatant was filtered through a 0.22 μm filter membrane, and 2 μl of desorption solution was injected into the UPLC–MS/MS system for analysis.

#### Standard curve.

A series of stock solutions containing imidacloprid and thiamethoxam in methanol were prepared. A calibration curve was plotted using 8 different concentrations (from 1 µg/ml to 100 µg/ml) of standards. All solutions were preserved in the freezer at −20 °C until use. The method was verified by determining the limit of detection (LOD), limit of quantitation (LOQ), linearity, and the matrix effect (ME). ME (%) was calculated based on the following equation (1) (Besil et al. 2017):


$$\begin{array}{l} ME\;\;\;(\%)=100\times \frac{S_{matrix}-S_{solvent}}{S_{solvent}}, \end{array}$$ (1)

where *S*_matrix_ and *S*_solvent_ are the slopes of the calibration curves in the matrix and pure solvent, respectively. An |ME| value of ≤20% is considered as a weak or no matrix effect; a value of 20% < |ME| ≤50% is considered as a moderate matrix effect; and an |ME| value of >50% is considered as a strong matrix effect. The relative standard deviation (RSD) of the 0–12 h stability test reflects the precision of the test method at 1 test concentration. The RSD of the repeated test reflects the precision of the method at each test concentration. The developed method was validated based on its linearity, ME, the LOQ, and the LOD.

### Statistical Analysis

The efficacy of insecticide treatment was evaluated 5, 10, 15, 20, 25, 30, and 45 DAT by analyzing the population densities of thrips using 2-way ANOVA (analysis of variance). Thrips densities in response to the same rates of application of both insecticides among different DAT and thrips densities among different insecticide treatments on the same DAT were analyzed by one-way ANOVA. Means were evaluated by Tukey’s HSD test at *P* < 0.05. The differences in the thrips density between each treatment of the different rates of both insecticides and that of the control were compared by *t*-tests using Dunnett’s procedure. Field data were analyzed using SPSS 16.0 software (SPSS Inc., Chicago, IL) (Chen et al. 2022). Linear regression was performed on the residual concentration–thrips density of imidacloprid and thiamethoxam in cowpea plants, and the fitting degree was determined by squares of correlation coefficients (*R*^2^). The log conversion of the residual concentration was conducted prior to analysis (Sharma and Singh 2014).

## Results

### Test of Correlation

**Fig. 1.**
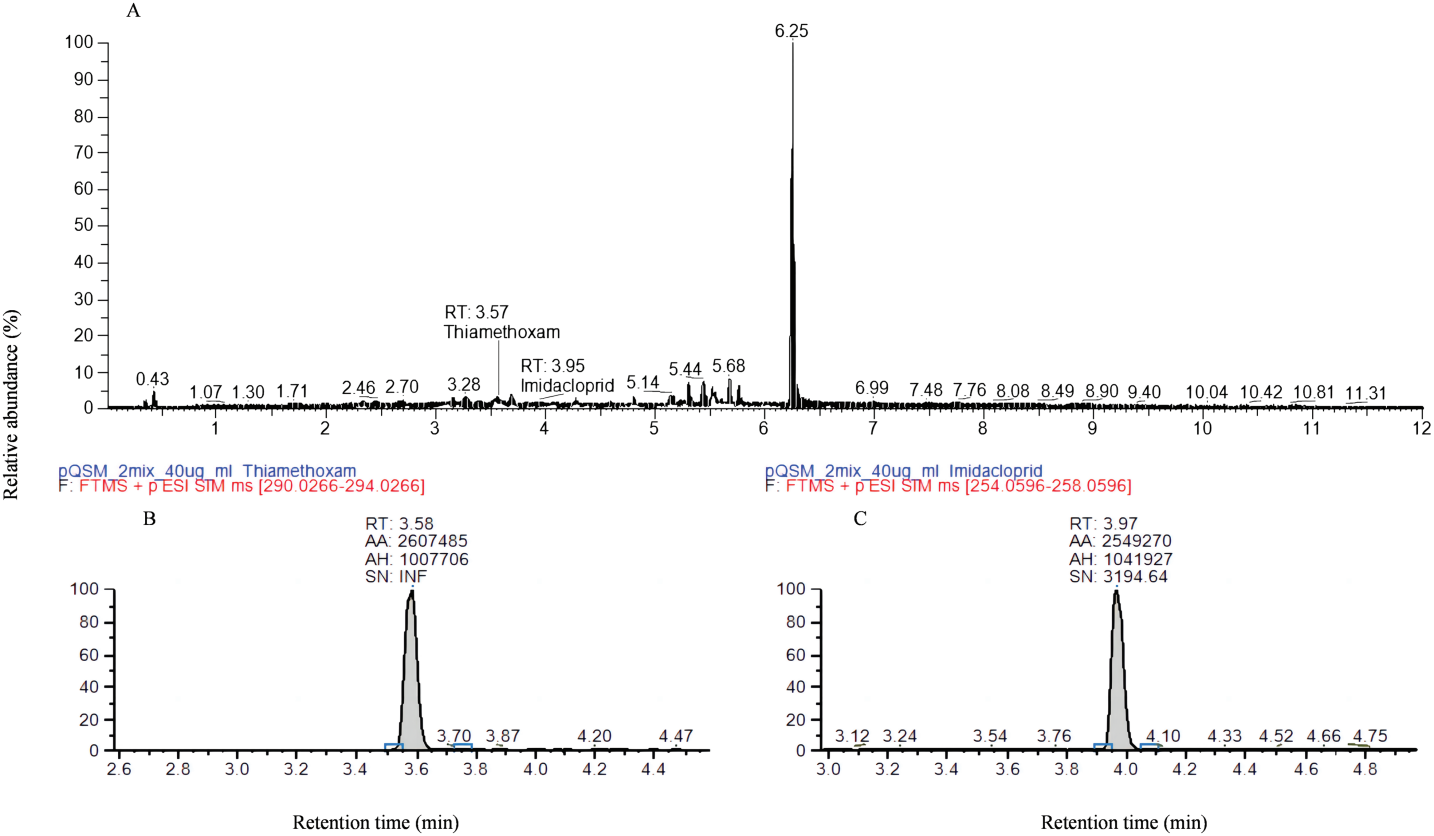
Chromatograms of (A) the standard mixture of thiamethoxam and imidacloprid, (B) thiamethoxam alone, and (C) imidacloprid alone.

The chromatographic peak area and concentrations of imidacloprid and thiamethoxam were linearly correlated in the range of 1–100 μg/ml. The correlation coefficients of the regression equations (imidacloprid: *y* = 6.762 × 10^4^*x* + 104497; thiamethoxam: *y* = 6.684 × 10^4^*x* + 106930) were high (imidacloprid: 0.9943; thiamethoxam: 0.9957), indicating that this method can be used for the qualitative and quantitative analysis of the 2 insecticides in cowpea and soil. The mean recoveries were 83.30–104.12% with RSDs of 0.97–8.29%. The LOD and LOQ for both insecticides ranged from 0.0004 to 0.0005 mg/kg and 0.010 to 0.013 mg/kg, respectively, in cowpea and soil (Table 1). The data in Table 1 indicate that the 2 insecticides differ slightly in their responses to different tissue sites, with MEs, and all less than 20%. The RSD results for 0–12 h for thiamethoxam and imidacloprid were 3.72 and 5.60%, respectively, and for the repeated test, the results were 3.97 and 5.80%, respectively. The total ion flow diagram of the mixed standard solution of the 2 insecticides and the ion flow diagram of each insecticide are shown in Fig. 1. The retention times of imidacloprid and thiamethoxam were 3.97 and 3.58 min, respectively.

**Table 1.** Calibration equations, correlation coefficients, and matrix effects of imidacloprid and thiamethoxam on cowpea (fruits, leaves) and soil

Compound	Matrices	Concentration (μg/ml)	Linear equation	Correlation	LOQs (mg/kg)	LODs (mg/kg)	ME (%)
Thiamethoxam	Acetonitrile	1−100	*y* = 6.762 × 10^4^*x* + 104497	*R* ^2^ = 0.9957			
	Fruits		*y* = 7.683 × 10^4^*x* + 17453	*R* ^2^ = 0.9971	0.010	0.0004	13.62
	Leaf		*y* = 7.647 × 10^4^*x* + 14561	*R* ^2^ = 0.9996	0.010	0.0004	13.09
	Soil		*y* = 7.109 × 10^4^*x* + 19263	*R* ^2^ = 0.9995	0.010	0.0004	5.13
Imidacloprid	Acetonitrile	1−100	*y* = 6.684 × 10^4^*x* + 106930	*R* ^2^ = 0.9943			
	Fruits		*y* = 6.097 × 10^4^*x* + 12721	*R* ^2^ = 0.9951	0.013	0.0005	−8.78
	Leaf		*y* = 6.681 × 10^4^*x* + 2317	*R* ^2^ = 0.9986	0.013	0.0005	−0.04
	Soil		*y* = 6.514 × 10^4^*x* + 5211	*R* ^2^ = 0.9998	0.013	0.0005	−2.54

LOQ: limit of quantification; LOD: limit of detection; ME: matrix effect.

### Efficacy of Imidacloprid and Thiamethoxam Against *M. usitatus* Applied by Root Irrigation

**Fig. 2.**
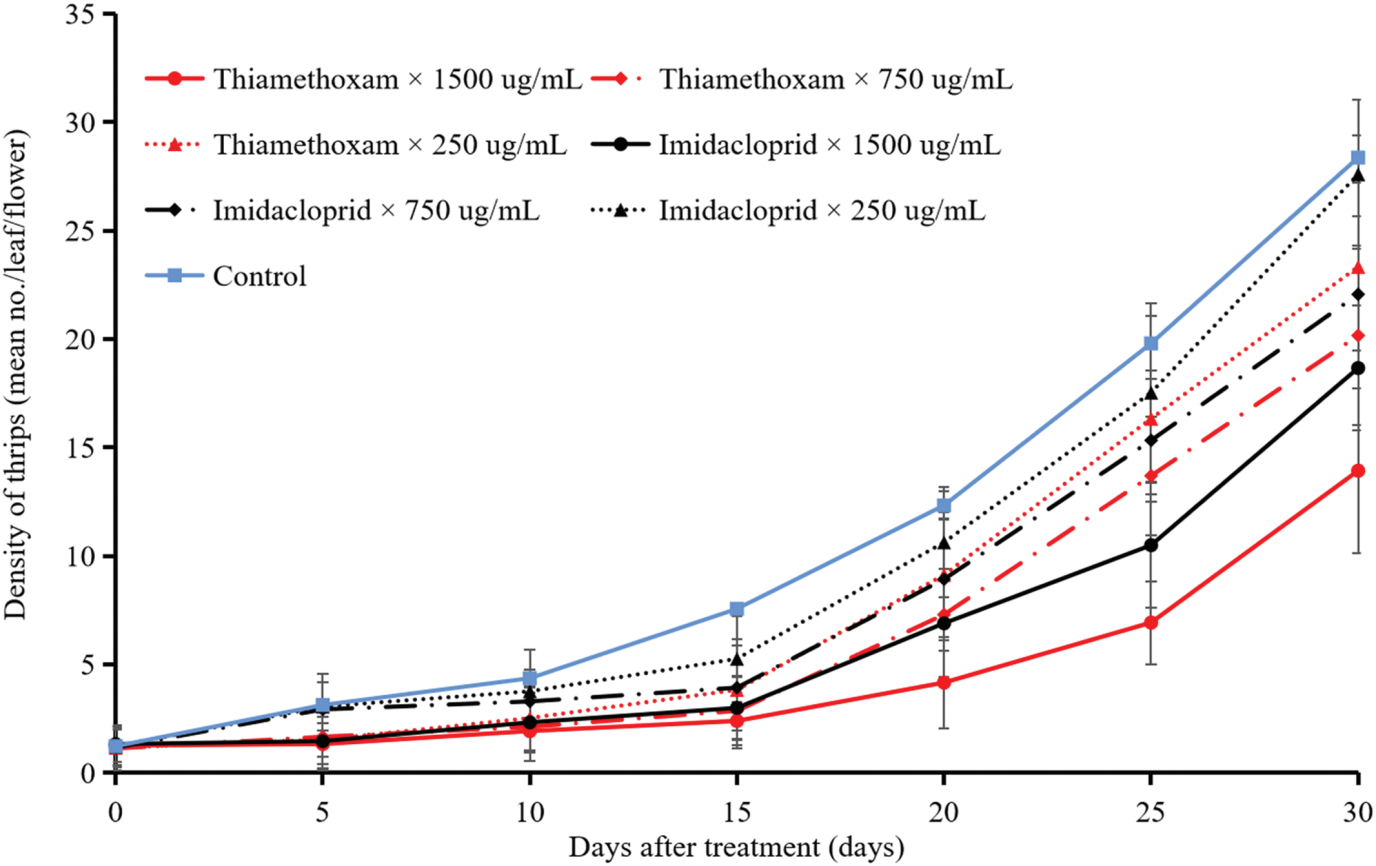
Densities of thrips in the field during the study period (mean number of thrips/leaf/flower). Values represent the means (±SE) of 3 replications.

The results of statistical analysis show that insecticides, DAT, and their interactions significantly affect the density of thrips (insecticides: *F* = 164.056; df = 6, 146; *P* < 0.001, DAT: *F* = 2,093.351; df = 6, 146; *P* < 0.001, interaction: *F* = 19.947; df = 36, 146; *P* < 0.001). The results showed that both imidacloprid and thiamethoxam significantly inhibited the population growth of thrips in the field, as indicated by the differences between the treatments and the untreated control (*t*-test, *P* < 0.05). The thrips densities under each treatment of both insecticides at the different DATs were significantly different (5 DAT: *F* = 23.783, df = 6, 20, *P* < 0.001; 10 DAT: *F* = 12.334, df = 6, 20, *P* < 0.001; 15 DAT: *F* = 25.130, df = 6, 20, *P* < 0.001; 20 DAT: *F* = 44.697, df = 6, 20, *P* < 0.001; 25 DAT: *F* = 35.196, df = 6, 20, *P* < 0.001; 30 DAT: *F* = 46.197, df = 6, 20, *P* < 0.001). Furthermore, Density of thrips increased for each increasing DAT (Table 2, Fig. 2). The results also showed that the thrips densities in each treatment of both insecticides differed significantly, with the lowest densities found in thiamethoxam 1,500 µg/ml at all DAT (*P* < 0.05) (Table 2). However, the thrips densities in each thiamethoxam treatment, as well as that in the maximum application rate (1,500 µg/ml) of imidacloprid did not differ significantly 5, 10, and 15 DAT (Table 2). Thrips abundance increased by 23.0-fold after 30 days in the control treatment, and by 10.9- to 22.4-fold in the insecticide treatments compared with the first sample. The thrips populations were significantly inhibited by both thiamethoxam and imidacloprid up to 25 and 20 DAT at the highest application rate, respectively, as indicated by thrips densities below the economic injury level of thrips damage for cowpea (7 thrips/flower [Nabirye et al. 2003]). In other words, the efficacies of both insecticides increased at increasing application rates during the whole sampling period. The lowest densities of thrips were found with thiamethoxam at the maximum rate of 1,500 µg/ml, followed by imidacloprid at the same application rate in all samples.

**Table 2.** Densities of thrips in the field during the study period expressed as mean number of thrips/leaf/flower

Date	Control	Thiamethoxam (µg/ml)			Imidacloprid (µg/ml)		
		1,500	750	250	1,500	750	250
31 Mar 2021 (pretreatment)	1.23 ± 0.93aF	1.27 ± 0.91aE	1.13 ± 1.00aE	1.17 ± 0.83aE	1.33 ± 0.84aE	1.25 ± 0.87aE	1.23 ± 0.97aF
5 Apr 2021	3.13 ± 1.17aE	1.33 ± 0.96bDE	1.67 ± 0.96bE	1.53 ± 1.33bE	1.47 ± 1.38bE	2.93 ± 1.68aDE	3.06 ± 1.11aE
10 Apr 2021	4.37 ± 1.40aE	1.93 ± 1.41cDE	2.13 ± 1.22cDE	2.53 ± 1.50cDE	2.33 ± 1.39cDE	3.30 ± 1.49bDE	3.76 ± 1.92abE
15 Apr 2021	7.57 ± 1.74aD	2.40 ± 1.30eD	2.87 ± 1.59deD	3.83 ± 2.35cdD	2.97 ± 1.47cdeD	3.93 ± 2.00cD	5.27 ± 2.01b D
20 Apr 2021	12.33 ± 3.19aC	4.17 ± 2.13eC	7.30 ± 1.68dC	9.13 ± 2.93cC	6.90 ± 2.54dC	8.93 ± 2.84cC	10.63 ± 2.58bC
25 Apr 2021	19.80 ± 3.56aB	6.93 ± 1.95fB	13.70 ± 2.78dB	16.33 ± 3.56bcB	10.50 ± 2.94eB	15.33 ± 2.87cB	17.53 ± 4.19bB
30 Apr 2021	28.37 ± 4.62aA	13.93 ± 3.85eA	20.83 ± 4.22cA	23.33 ± 3.92bA	18.67 ± 2.92dA	22.07 ± 3.64bcA	27.60 ± 3.44aA

Means (±SE) followed by the same lowercase letter in the same row indicate values that are not significantly different among the different insecticide treatments. Means (±SE) followed by the same uppercase letter in the same column indicate values that are not significantly different among the different days after treatments for the same tested insecticide treatment (by HSD test at *P* < 0.05).

### Imidacloprid and Thiamethoxam Residues in Cowpea and Soil

**Fig. 3.**
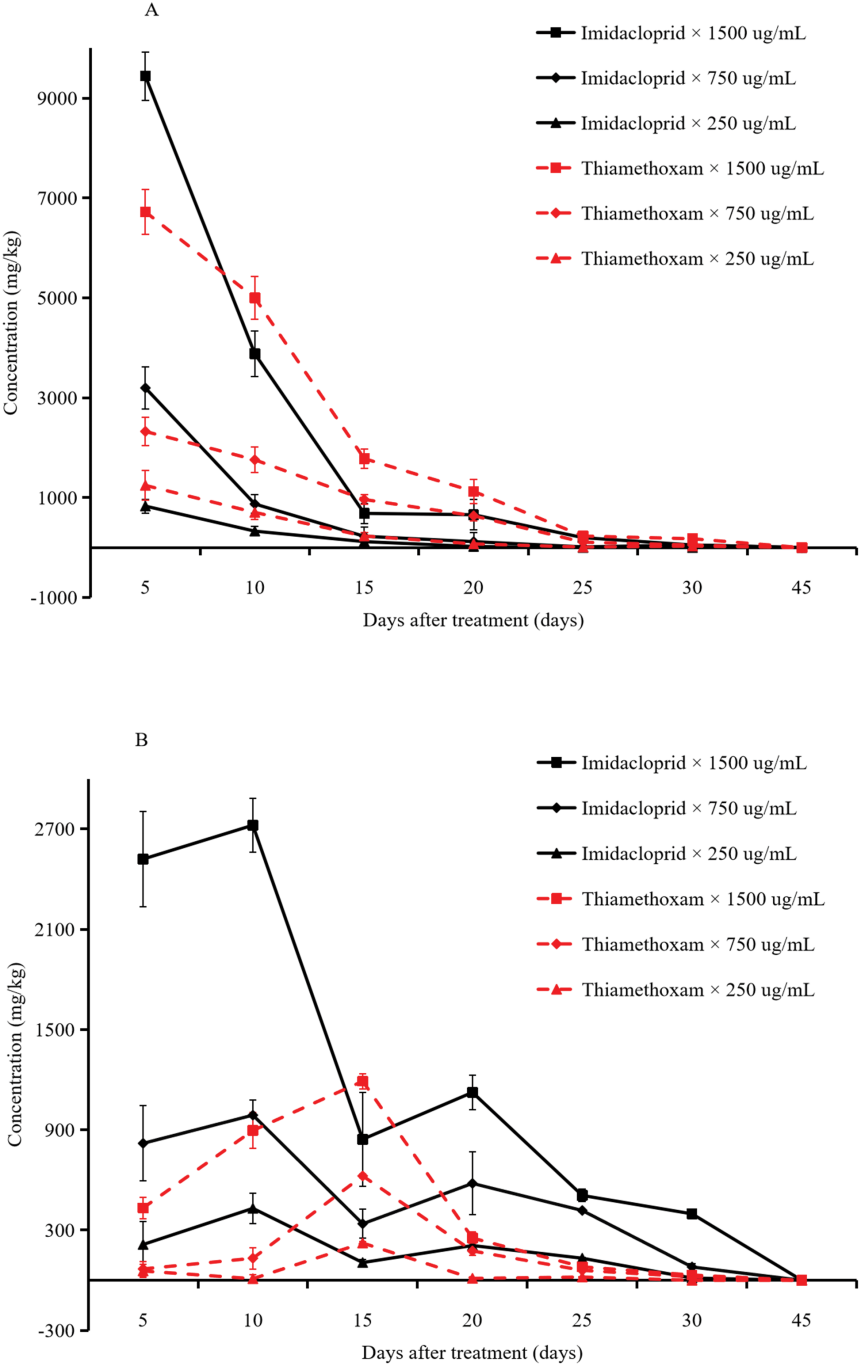
Residues of imidacloprid and thiamethoxam applied via root irrigation in (A) plants and (B) soil. Values represent the means (±SE) of 3 replications.

The residue levels of the target insecticides in cowpea plants (A) and soil (B) samples treated by root irrigation are shown in Fig. 3. The results show the detection of imidacloprid and thiamethoxam in cowpea plants until the penultimate sampling (30 DAT), with residue levels gradually decreasing over time. Five DAT, imidacloprid, and thiamethoxam were rapidly absorbed and translocated by cowpea plants, and the uptake and translocation rates increased as the insecticide concentrations increased. The maximum detection levels of imidacloprid and thiamethoxam were 9,442.05 and 6,722.83 mg/kg, respectively, which were found 5 DAT, at the insecticide application rate of 1,500 µg/ml (the maximum applied rate). Imidacloprid and thiamethoxam were rapidly decomposed in plants 15 DAT, and then their levels gradually stabilized. Thirty DAT, imidacloprid, and thiamethoxam applied at 1,500 µg/ml were detected at 51.64 and 177.18 mg/kg, and 0.00 and 30.17 mg/kg at 250 µg/ml, respectively (Fig. 3A). However, the residues of both insecticides were undetectable (<0.001 mg/kg) at 45 DAT.

Pesticide degradation trends in soil samples differed from those in plants, and they also differed between the 2 insecticides. The residue levels of imidacloprid fluctuated, with the maximum amount of pesticide detected 10 DAT, and then gradually decreased over time (Fig. 3B). Similar digestion dynamics were observed for thiamethoxam in soil, although residue levels fluctuated more during the first 15 DAT (Fig. 3B). The levels of insecticide residues from the high application rate were significantly higher than those at the low application rates. Thirty days after the root irrigation application of 1,500 µg/ml of both imidacloprid and thiamethoxam, 397.49 and 31.74 mg/kg were still detected, respectively. These results show that residues of imidacloprid and thiamethoxam remain in the soil for more than 30 days. Similar to residual dynamics in plants, no insecticide residues (<0.001 mg/kg) were detected in the last sample (45 DAT).

### Correlation of Imidacloprid and Thiamethoxam Residues in Cowpea Plants With the Density of Thrips

**Fig. 4.**
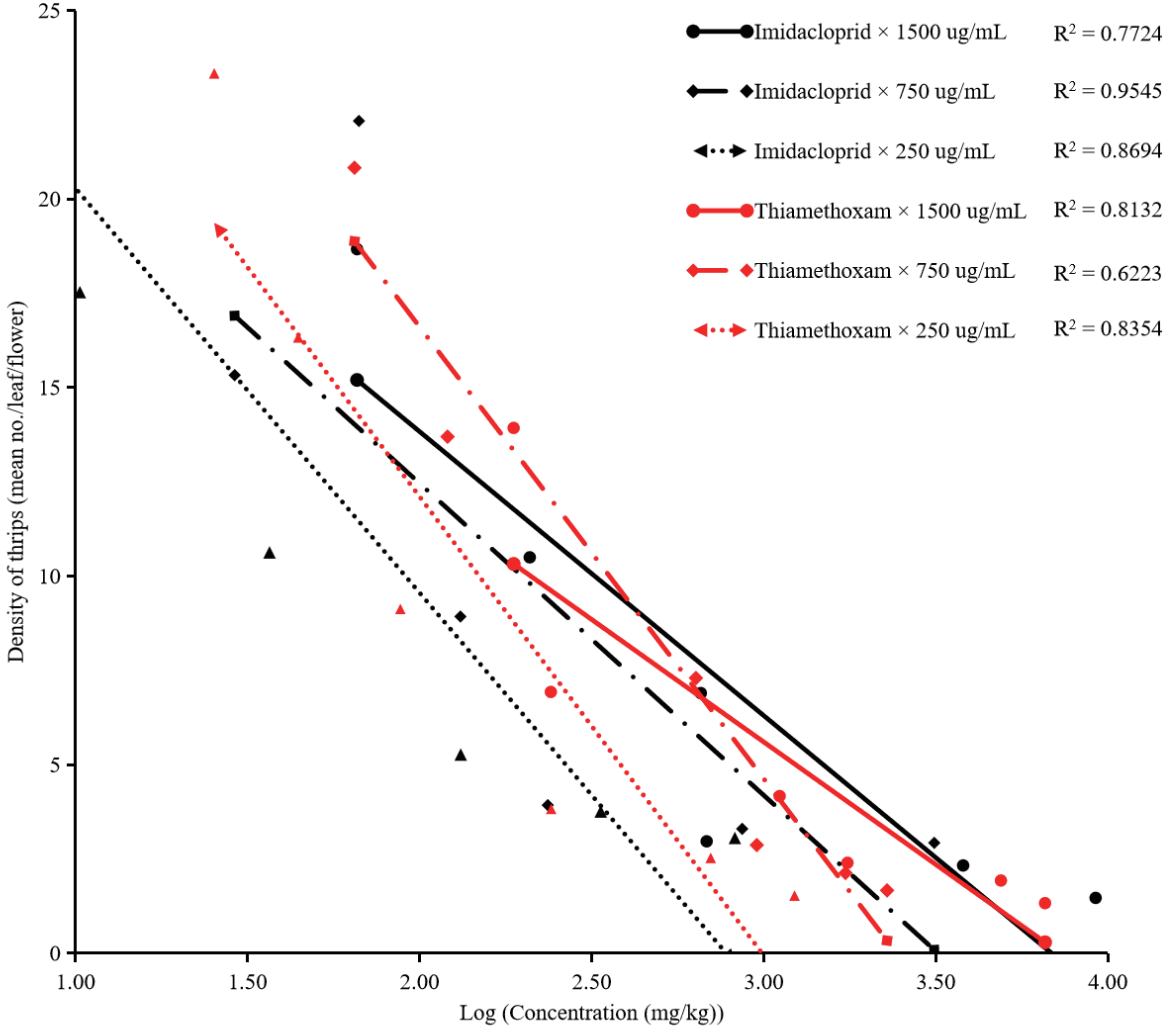
Linear relationship between log (concentration of insecticides) and density of thrips.

The density of thrips followed a linear relationship with the log (residue concentration) of the 2 insecticides (Fig. 4). The correlation coefficients (*R*^2^) between thiamethoxam residues applied at concentrations of 1,500, 750, and 250 µg/ml and thrips density were 0.8132, 0.6223, and 0.8354, respectively, indicating a dose–response relationship; while the *R*^2^ values between the imidacloprid residues and thrips densities were 0.7724, 0.9545, and 0.8694, respectively.

## Discussion

The systemic mechanism of NEOs provides theoretical evidence for their root application for the control of piercing–sucking insect pests. For example, root application of imidacloprid in *Bemisia tabaci* (Gennadius) (Hemiptera: Aleyrodidae) was more effective, as compared with seed or foliar applications (Abdel-razik 2019). In this study, we demonstrated that, except for the lowest application rate of imidacloprid, the various root irrigation application rates of imidacloprid and thiamethoxam were effective in controlling *M. usitatus* on cowpea for more than 25 days (Fig. 2). This prolonged activity may be due to the high pesticide residues remaining in cowpea after 30 days treatment (Fig. 3). Pesticide persistence is a double-edged sword. On one hand, the persistent effect (≥30 days) of imidacloprid and thiamethoxam results in thrips control, whereas on the other hand, pesticide residue levels exceed the maximum residual level (MRL) for cowpea. However, imidacloprid and thiamethoxam residues in pods and soil at the first cowpea harvest (45 DAT) were less than 0.001 mg/kg, which is significantly lower than the MRLs for cowpea in China (2 mg/kg for imidacloprid and 0.3 mg/kg for thiamethoxam), as well as that for the European Union (0.3 mg/kg for thiamethoxam) (Chen et al. 2021). Thus, these insecticides are safe for consumers.

Results of fluorescent-labeling show that imidacloprid is conducted downwards through the phloem sieve tubes and upwards mainly through vessels by transpiration (Zhu et al. 2021, 2022). The uptake and transport of insecticides in plants may also occur through seed or root pathways, which subsequently enable insecticides to reach other tissues within the plant (Tang et al. 2020). The transport rate is regulated by the physical and chemical properties of plants (Wang et al. 2021), which are, in turn, affected by abiotic factors such as water stress (Stamm et al. 2016). Imidacloprid and thiamethoxam are both absorbed and transported efficiently in plants, resulting in significantly higher accumulations of these insecticides in plant leaves as opposed to the roots (Ge et al. 2017). Insecticides that are swiftly absorbed and translocated to different plant parts subsequently interact with piercing–sucking pests (Elbert et al. 1991, Jeschke et al. 2011). These properties of NEOs indicate that root irrigation is an effective method for the control of crop diseases and insect pests (Stamm et al. 2016, Antu et al. 2017, Dhaliwal et al. 2017). Our study revealed that root application of imidacloprid and thiamethoxam resulted in rapid absorption and translocation to cowpea leaves, leading to a significant reduction in the thrips population within the first 2 wk after application (Fig. 3A), these findings are consistent with the absorption and transport characteristics of the 2 insecticides that have been observed on other crops (Stamm et al. 2016, Ge et al. 2017, Tang et al. 2020, Zhu et al. 2021). However, 30 days after root treatment of imidacloprid and thiamethoxam, they were still detected in cowpea leaves at levels of 7.64–66.59 and 25.29–187.46 mg/kg, respectively, which shows significantly longer persistence than that of foliar applications of NEOs (Liu et al. 2014, Chen et al. 2021, Reddy and Paul 2021, Wang et al. 2022) and in other crop systems (Abd-Ella 2014, Abdella et al. 2015, Nassar et al. 2015). Our results reveal different dissipation trends of the insecticides in soil and in plants. Furthermore, the insecticides tended to persist longer in the soil (Fig. 3B) at levels significantly greater than that of foliar applied insecticides (Liu et al. 2014).

Our field results demonstrate that cowpea plants treated with insecticides through root irrigation at the maximum application rate exhibited considerably lower thrips population densities over a prolonged period (≥25 days). These findings align with the levels of insecticide residues found in cowpea plants, which exceeded 200 mg/kg 25 DAT for insecticides applied at the maximum recommended rate. Moreover, these insecticides applied via root treatment are systematically distributed and remain active throughout the plant. The linear relationship between the insecticide residues in cowpea and the density of thrips strongly supports dose–response internal relation (Fig. 4). Furthermore, our findings indicate a considerable dose-effect relationship, i.e., a lower application rate resulted in a lower detection level and efficacy against thrips. A similar dose-effect relationship was found when NEOs were applied via root irrigation against tomato whitefly (Abdel-razik 2019). In general, the field efficacy of insecticides is closely related to the status of insecticide resistance. Our previous resistance monitoring results show that *M. usitatus* was sensitive to imidacloprid and thiamethoxam (Tang et al. 2015b), suggesting that these insecticides, applied via root irrigation, can exert control over 30 days for a long period of time by their residual toxicity.

In conclusion, this study shows the residual dynamics of 2 neonicotinoid insecticides (imidacloprid and thiamethoxam) in cowpea and soil, as well as their effects on 2 species of thrips in cowpea plants. The results reveal that the residues of root-applied imidacloprid and thiamethoxam persist in cowpea for more than 30 days (encompassing the entire pre-flowering period) during which they maintained the thrips population below the EIL (<7 thrips/plant). Thiamethoxam exhibited greater efficacy than imidacloprid. Imidacloprid and thiamethoxam residues were not detected in cowpea pods; thus, these 2 insecticides may be considered suitable for *M. usitatus* management while being safe for humans. We recommend using these insecticides as a part of an integrated approach toward thrips management of cowpea. Whenever feasible, imidacloprid and thiamethoxam should be applied via root irrigation at a dose not exceeding 1,500 µg/ml.
